# Transformer Performance for Chemical Reactions: Analysis of Different Predictive and Evaluation Scenarios

**DOI:** 10.1021/acs.jcim.2c01407

**Published:** 2023-03-23

**Authors:** Fernando Jaume-Santero, Alban Bornet, Alain Valery, Nona Naderi, David Vicente Alvarez, Dimitrios Proios, Anthony Yazdani, Colin Bournez, Thomas Fessard, Douglas Teodoro

**Affiliations:** † Department of Radiology and Medical Informatics, University of Geneva, 1205 Geneva, Switzerland; ‡ Geneva School of Business Administration, HES-SO University of Applied Sciences and Arts of Western Switzerland, 1227 Geneva, Switzerland; § SpiroChem AG, 4058 Basel, Switzerland; ∥ Swiss Institute of Bioinformatics, 1015 Lausanne, Switzerland

## Abstract

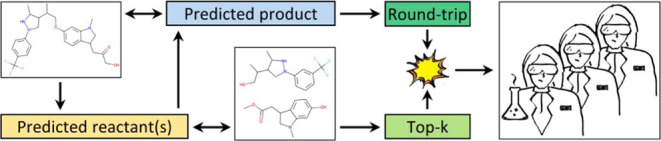

The prediction of chemical reaction pathways has been accelerated by the development of novel machine learning architectures based on the deep learning paradigm. In this context, deep neural networks initially designed for language translation have been used to accurately predict a wide range of chemical reactions. Among models suited for the task of language translation, the recently introduced molecular transformer reached impressive performance in terms of forward-synthesis and retrosynthesis predictions. In this study, we first present an analysis of the performance of transformer models for product, reactant, and reagent prediction tasks under different scenarios of data availability and data augmentation. We find that the impact of data augmentation depends on the prediction task and on the metric used to evaluate the model performance. Second, we probe the contribution of different combinations of input formats, tokenization schemes, and embedding strategies to model performance. We find that less stable input settings generally lead to better performance. Lastly, we validate the superiority of round-trip accuracy over simpler evaluation metrics, such as top-*k* accuracy, using a committee of human experts and show a strong agreement for predictions that pass the round-trip test. This demonstrates the usefulness of more elaborate metrics in complex predictive scenarios and highlights the limitations of direct comparisons to a predefined database, which may include a limited number of chemical reaction pathways.

## Introduction

The synthesis of chemical compounds allows for the development of new pharmaceutical drugs that improve patient conditions while minimizing side effects.^1,2^ However, due to the large number of possible molecular combinations that could produce valid drugs (up to ∼10^60^), searching for the safest and most efficient compounds is a challenging problem.^3−5^ Testing all possible combinations of chemical precursors is intractable. For this reason, research techniques based on artificial intelligence and machine learning have been developed and proved successful in the task of chemical reaction prediction.^6−8^

In the last few years, the use of artificial intelligence in the field of biochemistry has started to accelerate the discovery of new compounds and to increase the variety of commercial drugs while decreasing their research costs.^9−12^ Some of the areas enhanced by machine learning algorithms are molecule generation,^13,14^ chemical reaction optimization,^15−17^ direct reaction prediction,^18−20^ and retrosynthesis prediction.^21−25^ Previous studies in machine learning for chemistry have proposed a wide range of models based on different branches of artificial intelligence such as evolutionary algorithms,^26^ unsupervised learning,^27,28^ graph neural networks,^19,29^ and natural language processing (NLP).^15−18^

In particular, neural machine translators (NMT) are a class of NLP models trained with large sets of documents to translate sequences of text from one language to another. NMT models are trained with text-like data, in which sequences of tokens, following specific syntactic rules and encoding specific semantic relationships, are provided to their input layer. To apply NMT models to the case of chemistry, different string-based molecular representations developed in the last few decades can be used^30−34^ (for a review, see ref (35)). The task given to the models is to translate a set of target molecule strings (e.g., reactants) to their corresponding target molecules (e.g., product). In this context, language models are trained to capture the underlying relationships between molecular substructures present in the reaction strings and learn enhanced token representations which may be used in downstream predictive tasks.^36,37^ The most popular string format in the field is the simplified molecular-input line-entry system (SMILES),^30^ in which atoms and bonds are listed following the different branches of a molecular graph and cycle loops are indexed along the string. Thanks to the procedure designed by Lowe for extracting chemical structures from patents,^38,39^ millions of chemical reactions from the United States Patent and Trademark Office (USPTO) have been recovered and published as SMILES sequences.^40^

There are several model architectures suitable for machine translation, such as recurrent neural networks (RNNs),^41^ long short-term-memory networks (LSTMs),^42^ and transformers.^43^ While RNNs and LSTMs process information sequentially, i.e., sentences are processed token by token, transformers present a self-attention mechanism that allows whole input sequences to be processed at once. Transformers have an encoder–decoder structure and combine positional encoding with multiple attention heads to determine how different input tokens relate to each other.^36,37^ One of the main advantages of using transformers for sequence processing is that it prevents the loss of contextual information, as often observed in recurrent models.^43^ Moreover, in the model computations, the maximum path length between tokens does not depend on their distance, which provides an advantage for long-range dependency modeling.^44^

Among NMT models, transformers obtain the most outstanding results for the task of chemical reaction prediction. Stemming from the seminal work of Schwaller and colleagues^18^ who introduced the molecular transformer, numerous studies have shown the high potential of attention-based models for the task of direct product prediction,^18,22,24^ single-step^22,24,45−48^ and multi-step^23,49^ retrosynthesis, reaction classification,^50^ and atom mapping.^28^ In the following, we state the relevant research advances related to the use of transformers for chemical reaction prediction and point out existing knowledge gaps, which motivated the current study.

First, by shuffling the first atom listed in the SMILES string and the direction of graph enumerations, it is possible to build many non-canonical SMILES strings for a single molecule.^24^ This allows us to perform almost unlimited data augmentation with chemical reactions. Yet, data augmentation might have limits and may impact model performance differently, depending on the task and evaluation metric. Previous studies have debated the importance of data augmentation for different predictive scenarios.^18,24,51^ For example, it was shown that retrosynthesis prediction benefits more from data augmentation than direct product prediction in terms of top-*k* accuracy.^24^ This raises the question as to how different combinations of tasks, data augmentation levels, and evaluation metrics contribute to model performance.

Second, another increasingly popular string format for molecules is SELFIES, standing for Self-Referencing Embedded Strings.^31^ SMILES can produce strings that do not correspond to any valid molecule (actually, most random sequences of SMILES symbols are invalid), and this may be seen as a weakness.^35^ SELFIES solve this issue by producing a syntax in which all generated strings are valid chemical molecules. Any valid SMILES string can be mapped to a SELFIES string without losing chemical information. However, although SELFIES is built to improve the quality of the input molecules as well as the stability of generated reactions, a robust syntax may restrict the regions of the data space that are explored by the model during training.

Third, to have a tractable representation space, language models require sentences to be parsed into smaller sets of characters, i.e., tokens. Different NLP tokenization strategies exist, such as character-based encoding, byte-pair encoding,^52^ word-piece,^53^ or sentence-piece,^54^ some of which are better suited for the task, language, and syntax given to the model. In the case of chemistry, the most common one is atom-level tokenization, where each token represents an atom, a type of chemical bond, or a closed loop in an atom chain. Considering reactions as sentences and molecules as words, this tokenization strategy is conceptually close to a character-based encoding. A potential limitation of this strategy is that it requires information carried by tokens to heavily rely on their context.^55^ In consequence, a lot of data are required to build useful context-independent representations of atoms. Conversely, byte-pair encoding joins atoms and bonds that are consistently repeated over the dataset, forming tokens that represent frequent molecular substructures,^56^ instead of representing all atoms separately. By recognizing these substructures, tokenizers can capture close-range atomic relationships within the molecules and transfer them into the predictive model, without requiring the model to learn them by itself. However, they also carry a higher computational cost associated with the combinatorially large number of molecule substructures.

Fourth, a popular technique to increase model performance is to provide pre-trained knowledge to the model at training time. Instead of learning the parameters of its input layer from scratch, a language model can use the output of a different model as a set of static features. Pre-trained token embeddings, when used as an underlying input representation, were shown to improve performance in various NLP tasks.^57,58^ In the case of chemistry, simple language models, such as word2vec,^59^ can be trained beforehand with a large set of molecules to ensure that the trained model is always aware of semantic and syntactic relationships between atoms and bonds inside molecule strings. This input strategy makes sure that chemical knowledge is present in the input representation of the model but also restricts its freedom, since the parameters of the input layer are kept fixed during training.

Arguably, SELFIES format, BPE tokenization, and pre-trained embeddings can be considered more stable and robust input settings than SMILES format, atom-level tokenization, and embeddings trained from scratch, respectively. Indeed, the former settings include pre-determined knowledge that the model should not discover by itself during training, which ensures more stable representations. For example, using SELFIES relieves the model from learning to generate valid molecules. In other words, during training, SELFIES shapes the loss landscape in the model parameter space and offers a restricted set of paths that the model can exploit to reach its goals in a robust way. Similarly, pre-trained embeddings and BPE tokenization restrict the regions of parameters that the model can explore, by ensuring that the representations of input tokens are chemically valid.

Conversely, these input settings may reduce the diversity of molecules that can be represented and generated by the model during and after training. In other words, these input settings transfer less expressivity to the trained model. For example, the SELFIES format only allows the generation of valid molecules, which may prevent the model from making insightful mistakes during training. Similarly, a model trained with BPE tokenization can only produce molecules out of specific patterns that were identified prior to training and hence cannot extend its predictions to previously unseen patterns. This may restrict the generalization capabilities of the model and/or lead to local loss minima during training. For these reasons, testing all combinations of the mentioned input settings enables us to compare the impact of expressivity and stability to model performance. There is a trade-off between model stability and expressivity,^60^ and which one provides the best performance for chemical reaction prediction remains an open question.

Finally, it has been pointed out that simple evaluation metrics such as top-*k* accuracy are not well-suited to evaluate model performance for retrosynthesis.^23,24,61^ Indeed, in most cases, several reactants must be predicted. In addition, several sets of reactants might lead to the same product, and the target reactants of the training and testing datasets represent only one possibility. New metrics that address these problems were proposed, one of them being the round-trip accuracy.^23^ Round-trip evaluates the performance of a retrosynthesis prediction model by computing the proportion of model predictions (i.e., reactants) that lead to the original product, once fed to a trained forward-synthesis prediction model. Round-trip analysis can be seen as asking a chemist expert to judge whether the predicted reactants could lead to the desired product. It was shown that the molecular transformer reaches higher round-trip accuracy than top-1 accuracy,^23^ which suggests that the model can correctly identify alternative retrosynthetic pathways. However, it is still possible that the round-trip test produces false positives, i.e., the forward prediction model recovers the correct product fortuitously from a bad set of predicted reactants. Hence, to confirm the superiority of round-trip analysis over simple metrics, it is crucial to determine whether it correlates with human expert judgements, especially in cases where simple metrics do not agree with round-trip accuracy.

The main contributions of the current study can be summarized as follows:

- First, we present an analysis of the performance of the molecular transformer for product, reactant, and reagent prediction tasks, under different scenarios of data availability and data augmentation. We show that the impact of data augmentation and adding reagent information depends on the prediction task and on the metric used to evaluate model performance.
- Second, we evaluate the impact of input stability and expressivity to model performance by using different input formats (SMILES vs SELFIES), tokenization strategies (atom-level vs byte-pair encoding), and input embeddings (learnt vs pre-trained). We show that more expressive input schemes generally lead to better performance.
- Lastly, a committee of human experts validated round-trip analyses for predictions that led to divergent evaluations as compared to top-1 accuracy. We quantitatively demonstrate that the model can identify alternative reaction pathways: for most predictions where round-trip and top-1 accuracies disagree, human experts are on the round-trip side. To the best of our knowledge, this is the first time this new metric is confronted to the judgment of a large pool of chemistry experts.

## Methodology

### 2.1 Reaction Prediction Model

**Figure 1:**
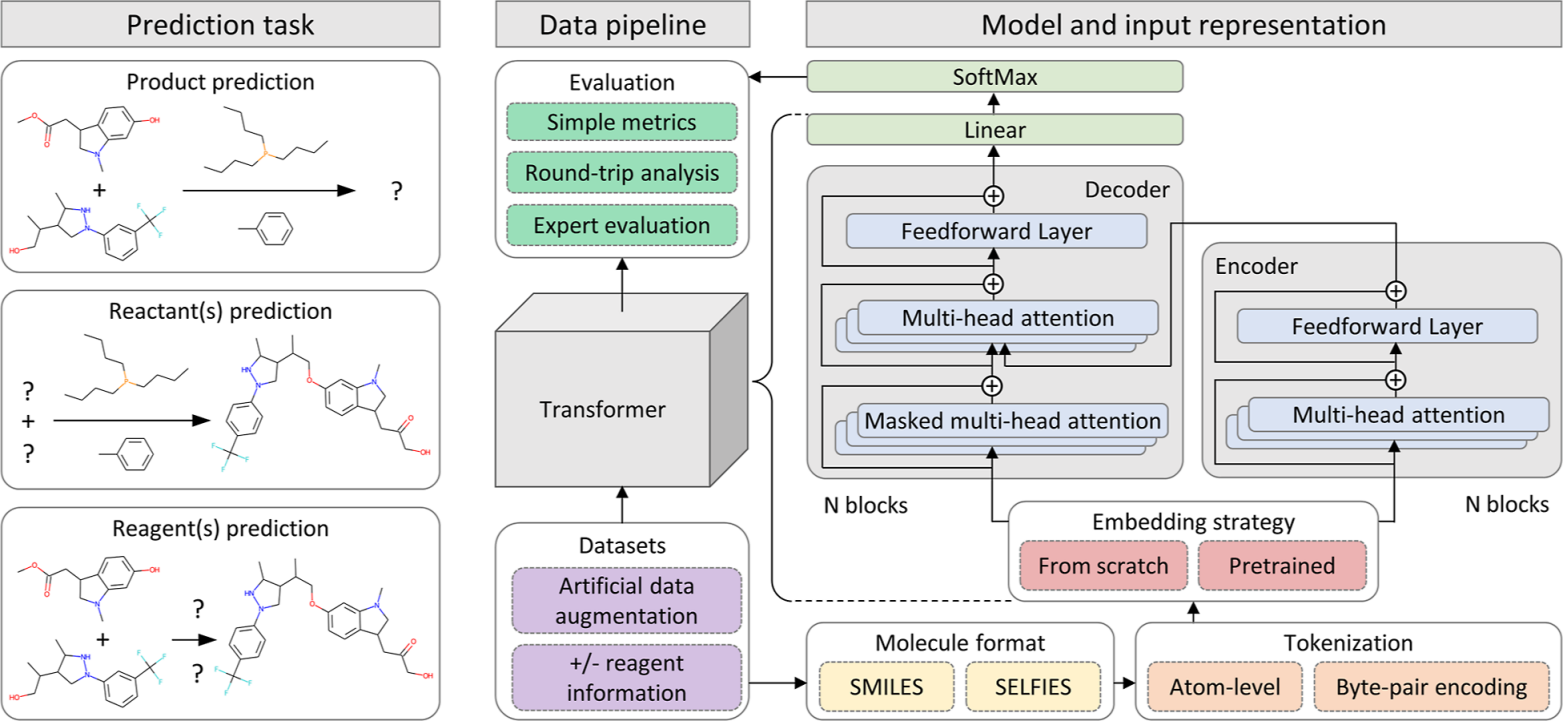
Product, reactant, and reagent prediction models were trained with different combinations of input settings. Dashed boxes represent interchangeable configurations of data augmentation, tokenization and embedding strategies, molecule formats, and evaluation metrics.

We used the molecular transformer^18^ for the predictive scenarios presented in this study (Figure 1). This model was originally set up in the OpenNMT ecosystem,^62^ which we used here (version 2.1). The molecular transformer has a four-layer encoder–decoder structure with an embedding dimension of 256, a feedforward dimension of 2048, eight self-attention heads with scaled dot-product, and uses the softmax function as the global attention operation, for a total of 11.7 M parameters. All model predictions were generated using the beam search algorithm with a beam size of 10.^24^ Model hyper-parameters were fixed in all experiments.

Figure 1 summarizes the predictive scenarios that we used in our experiments. We evaluated model performance using different datasets (purple boxes), input formats, tokenization schemes, embedding strategies (yellow, orange, and red boxes), and evaluation metrics (dark green boxes). The different experiments run in this study are detailed in the next subsections.

Throughout these experiments, the model was trained with a batch size of 4096 tokens, on one of the following tasks:

- Product prediction: the model must predict the product of the reaction, given its reactants (with/without reagents).
- Reactant prediction: the model must predict all reactants, given the product (with/without reagents) of the reaction. Note that, although including reagent information in a reactant prediction task is often an unrealistic scenario in practice, it is still interesting to evaluate its impact on model performance, as it represents a very favorable information scenario. Besides, a model trained in these settings might be useful when only a limited set of reagents are available. We also evaluated whether the model could predict at least one of the reactants of the reaction (lenient-reactant prediction).
- Reagent prediction: the model must predict all reagents, given the reactants and the product of the reaction. This task investigates whether the molecular transformer can anticipate optimal reaction conditions.

Since the molecular transformer was trained on differently sized datasets and with different input settings, we let the number of training steps be determined by early stopping, with a patience of 10, in order to prevent over- or under-fitting. More specifically, training was stopped whenever neither token-level prediction accuracy nor perplexity improved in the scope of 10 validation epochs. A validation epoch was run every 10k steps multiplied by the level of data augmentation.

### 2.2 Reaction Datasets and Data Augmentation

We used the USPTO-MIT dataset which, after removing inconsistencies and duplicates from the original dataset of Lowe,^38^ contains 480k valid reactions.^63^ These reactions are divided into three datasets of 410k, 30k, and 40k reactions for training, validation, and testing, respectively.^64^ To identify products, reactants, and reagents in reactions, we used the work of ref (65), which separated reagents from reactants in USPTO-MIT using atom mappings. We then built the source, i.e., the input molecule(s), and the target, i.e., the molecule(s) to predict, for each task presented in Section 2.1. When a model was trained and evaluated with reagent information, it was present in the training, validation, and testing datasets. As in the original work of the molecular transformer,^18^ we did not separate reactants and reagents using a special token when they appeared together in the model input (e.g., for product prediction). Instead, all molecules were separated by the same token (“.”), which was part of the model vocabulary. To evaluate model performance, in addition to the USPTO-MIT testing dataset, we also used USPTO-50k,^66^ whenever possible.

To analyze the impact of data augmentation on model performance, we augmented the training dataset of each task presented in Section 2.1. We created alternative versions of chemical reactions by using non-canonical SMILES notation and random permutations of reactants and reagents. We only performed data augmentation on the source input of the reactions (i.e., not the target). This led to five different versions of the training dataset for each task, containing 1, 2, 5, 10, and 20 times the size of the original data. In the first experiment, we simply compared the effect of data augmentation for each task described in Section 2.1. The results of this experiment are presented in Section 3.1.

In another experiment, we performed a detailed analysis of the impact of data augmentation on model performance for the reactant prediction task. We evaluated the models using two different metrics, namely, top-*k* accuracy and round-trip accuracy, a measure that is more suited for retrosynthesis scenarios (see Section 2.4 for more details about all evaluation metrics that were used in this work). We used the reactant prediction models trained in Section 3.1, i.e., with any level of data augmentation and including reagent information or not. We evaluated these models on both the USPTO-MIT and USPTO-50k test datasets. To perform the round-trip analysis, we matched the product prediction and reactant prediction models. For example, to evaluate the reactant prediction model that was trained without reagent information and with 5-fold data augmentation, we used the product prediction model that was trained without reagent information and with 5-fold data augmentation. The results of this experiment are presented in Section 3.2.

Finally, we performed a detailed analysis of model performance for reagent prediction. We divided the testing dataset in different subsets of samples, binning reactions by the number of reagents they contain. We computed precision, recall, and f1-score at the molecule-prediction level (see Section 2.4 for more details) to reveal the types of error the model was experiencing for different numbers of predicted reagents. We evaluated the reagent prediction models trained in Section 3.1. Since the USPTO-50k dataset does not include reagents, we only performed this analysis with the USPTO-MIT testing dataset. The results of this analysis are presented in Section 3.3.

### 2.3 Input Format, Tokenization, and Embedding

In this set of experiments, we trained and evaluated the molecular transformer on all tasks presented in Section 2.1, using all possible combinations of the following molecule formats, input embedding strategies, and tokenization schemes.

- Input format: the format of the molecules could either be SMILES or SELFIES. To generate the SELFIES datasets, we encoded the corresponding SMILES reactions using the code of ref (31). In very rare cases, some molecules (included in Table S9, Supporting Information) could not be encoded, in which case they were replaced by the token “?”.
- Tokenization scheme: reactions were parsed either by atom-based or byte-pair encoding (BPE). For BPE, we used the SMILES pair encoding (SPE) algorithm^56^ to identify the most frequent substrings present in the training dataset. We applied the same algorithm to identify frequent SELFIES substrings. To build the vocabularies, we only included substrings that occurred at least 2000 times in the training dataset. This led to vocabularies of around 1500 tokens, 10 times the size of the corresponding atom-level vocabularies.
- Input embedding strategy: the model could either learn the parameters of its input embedding layer from scratch (i.e., during training) or use a pre-trained input embedding layer. In the latter case, the input embedding layer was replaced by the static output of word2vec^59^ and frozen during training. The word2vec model (embedding size: 256 and window size: 5) was trained using a corpus of single (SMILES or SELFIES) molecules extracted from the training dataset and parsed by the selected tokenization scheme (i.e., atom-level or BPE).

The trained models were evaluated both with the USPTO-MIT testing dataset and USPTO-50k. The results of these experiments are presented in Section 3.4.

### 2.4 Evaluation Metrics

We used different metrics to evaluate model performance.

- Standard metric: to compare our results to the existing literature, we used top-*k* accuracy. An exact match with the target was imposed, after canonicalizing the prediction of the model and the target molecules. More precisely, to consider a model prediction as a hit, all target molecules must be present in the molecules predicted by the model, and no molecule predicted by the model must be absent from the set of target molecules. Then, top-*k* accuracy was defined as the proportion of target samples of the testing dataset for which at least one hit was present in the corresponding top-*k* model predictions.
- Round-trip accuracy:^23^ for the reactant prediction task, we fed the top-1 predictions of any reactant prediction model to the corresponding product prediction model (i.e., the model that was trained with the same amount of data augmentation and under the same conditions of reagent availability). When evaluating the reactant prediction model that was trained with reagent information, the input of the product prediction model was the concatenation of the predicted reactants and the true reagents of the reaction. Round-trip accuracy was defined as the proportion of reaction samples from the testing dataset for which the top-1 prediction of the product prediction model matched the input of the reactant prediction model.
- Precision, recall, and f1-score at rank *k*: for the reagent prediction task, we also evaluated model performance at the molecule level, since the number of target reagents varies a lot across samples (which means top-*k* accuracy might not be the best metric to assess model performance). For each test sample, we considered the set of N unique molecules present in the top-*k* model predictions. The true positive score (*T*) was defined as the number of predicted molecules in this set that matched any of the *M* corresponding target molecules. Precision (*P*), recall (*R*), and f1-score (*F*) were computed as *P* = *T*/*N*, *R* = *T*/*M*, and *F* = 2*PR*/(*P* + *R*), respectively.
- Expert validation: for the reactant prediction task, all reactions for which round-trip analysis produced a positive result, but simple accuracy did not, were independently analyzed by two groups of 10 experts each. One group was composed of experts with a master’s degree and the other one of experts with a PhD degree, both in the field of chemistry. Every reaction was seen exactly once by one master and one PhD. Experts were asked to associate one of the three possible scores to each predicted reaction: wrong, correct, or semi-correct. “Wrong” meant that the proposed reaction was not physically consistent. “Correct” meant that the predicted reactants offered a valid alternative pathway to the ones included in the USPTO-MIT dataset. “Semi-correct” meant that the reaction was physically consistent but did not feature the expected outcome, i.e., predicted residuals that would not usually be considered. The results of the expert validation experiment are presented in Section 3.5.

## Results and Discussion

### 3.1 Reaction Prediction Accuracy and Data Augmentation

**Figure 2:**
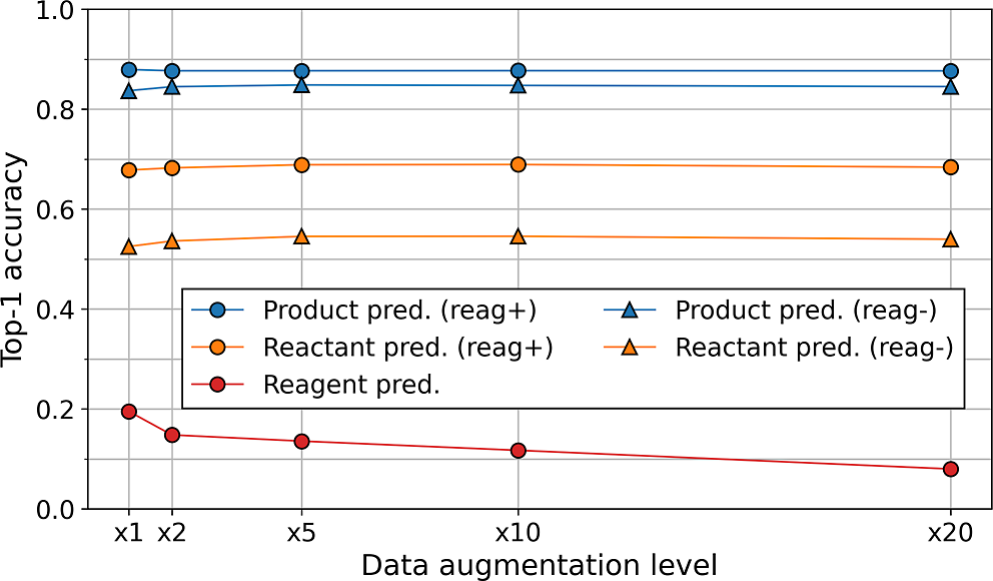
Effect of data augmentation on top-1 accuracy, computed with the USPTO-MIT testing dataset (“reag+”—reagent information included, and “reag–“—reagent information not included).

The molecular transformer model was trained on each prediction task described in Section 2.1, using the data augmentation schemes described in Section 2.2. Figure 2 shows the top-1 accuracy computed on the USPTO-MIT testing dataset, for all combinations of task and data augmentation. Results for top-*k* accuracy (*k* > 1) and using the USPTO-50k dataset are shown in Figures S1–S8 (Supporting Information).

For the product prediction task (Figure 2, blue), data augmentation has a minor impact on model performance and only when reagent information is not included (1% improvement, from 84 to 85% top-1 accuracy). When reagent information is included, top-1 accuracy stays around 88% for any level of data augmentation. These results are similar to those presented in the original work of the molecular transformer.^18^ For comparison, Tetko and colleagues^24^ used up to 100-fold data augmentation on the USPTO-MIT dataset, augmenting both source and target inputs, and product prediction top-1 accuracy increased up to 91%.

For the reactant prediction task, data augmentation has more impact than for product prediction (Figure 2, yellow). Model performance increases from 52 to 55% top-1 accuracy when reagent information is not included and from 68 to 69% when it is. Note that we do not use the USPTO-50k dataset to train our models, but the larger USPTO-MIT dataset. This explains the higher performance than what is usually reported for retrosynthesis prediction accuracy without reagent information (between 42 and 54% when trained with the USPTO-50k dataset, depending on whether reaction class information is provided as the input^24^). A more detailed analysis of reactant prediction is carried out in Subsection 3.2.

When using the USPTO-50k dataset to evaluate product and reactant prediction performance, data augmentation has a similar but less consistent effect on top-*k* accuracy (Figures S1–S8, Supporting Information). Surprisingly, models trained without reagent information reach higher performance as compared to when the USPTO-MIT testing dataset is used for evaluation. The product prediction model improves from 86 to 87% top-1 accuracy, and the reactant prediction model improves from 57 to 59% top-1 accuracy (Figure S5, Supporting Information). For comparison, Tetko and colleagues^24^ trained their model for reactant prediction on the USPTO-50k dataset, augmenting both source and target samples by 100-fold and reported an increase in top-1 accuracy from 48 to 54%. This is lower but still comparable to the performance we obtain using the whole USPTO-MIT data for training. Models trained with reagent information reach lower performance (up to 80 and 38% top-1 accuracy for the product and reactant prediction tasks, respectively) as compared to when the USPTO-MIT dataset is used for evaluation. We attribute this poorer performance to the absence of reagent information in the USPTO-50k dataset.

For the reagent prediction task, results show a significant drop in performance compared to the other tasks. Top-1 accuracy only reaches up to 20% (Figure 2, red). We attribute this poor performance to the larger and more variable number of reagents per reaction. Besides, reagent atoms, unlike reactants, cannot be mapped to the product atoms. Surprisingly, data augmentation has a detrimental effect on model performance. A possible reason is that both reactants and products appear in the source input in the reagent prediction task. Permuting molecules might be too confusing for the model, since it becomes very challenging to distinguish the product from the reactants and, hence, to identify the type of reaction taking place. A more successful strategy could have been to keep the order in which molecules appear in the reaction when augmenting the data. A more detailed analysis of reagent predictions is carried out in Subsection 3.3.

### 3.2 Analysis of Reactant Predictions

**Figure 3:**
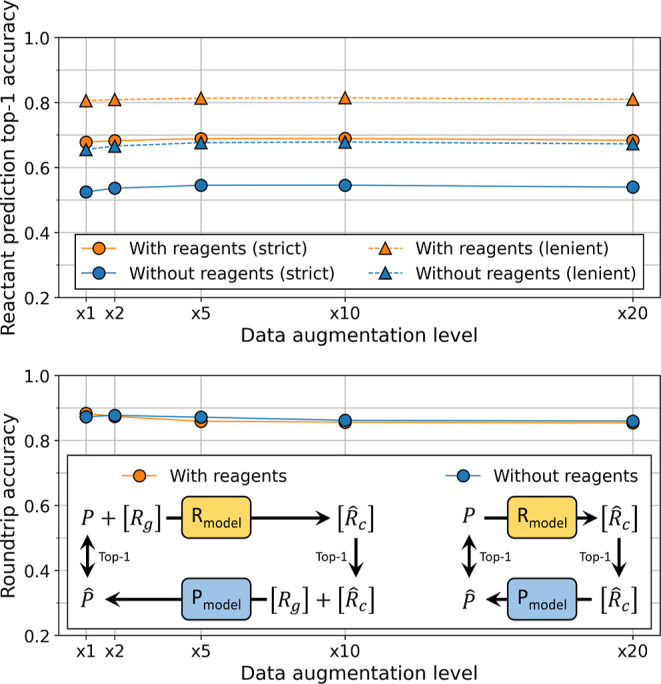
Top-1 and round-trip accuracy for the reactant prediction task, using the USPTO-MIT testing dataset, for different levels of data augmentation. Top. Top-1 accuracy. “Strict” requires an exact match between the model prediction and the target. “Lenient” requires that at least one molecule predicted by the model matches a target molecule. Bottom. Round-trip accuracy. The diagram shows how round-trip accuracy was computed. When reagents were part of the datasets, the true reagents were added to the predicted reactants before being sent to the product prediction model. *P*—true product, [*R*_c_]—true reactant(s), [*R*_g_]—true reagent(s), *P̂*—predicted product, and —predicted reactant(s).

To further investigate the performance of the molecular transformer for the task of reactant prediction, we evaluated the reactant prediction models trained in Section 3.1 with round-trip accuracy. Figure 3 shows top-1 and round-trip accuracy for the reactants and lenient-reactant prediction tasks, for all data augmentation levels. Reactant prediction accuracy is consistently improved by around 15% when reagent information is included, independent of the level of data augmentation (Figure 3, top, blue vs orange). When a lenient match is used to compute top-1 accuracy, model performance also increases by around 15% (Figure 3, top, triangles vs circles).

Regarding round-trip accuracy, model performance (up to 88%, Figure 3, bottom) is higher than top-1 accuracy. Round-trip accuracy even outperforms top-10 accuracy (86%, Figure S4, Supporting Information). This suggests that the reactant prediction model produces valid sets or reactants, even if they do not appear in the testing dataset. Remarkably, data augmentation and reagent information no longer affect model performance. Round-trip accuracy does not depend on whether reagent information is provided to the models (Figure 3, bottom, blue vs orange), and performance slightly decreases with data augmentation. The best performance is observed with un-augmented data when reagent information is included (88%) and with 2-fold data augmentation when it is not (87%). These results can be compared to ref (23), in which 70 to 81% round-trip accuracy was obtained, depending on the data used to train and test the model. Note that the task used in their study was to predict both reactants and reagents from the products, which may explain the higher performance obtained in our case.

We also measured round-trip accuracy with the USPTO-50k dataset. Although top-1 accuracy is higher when evaluated with USPTO-50k than with the USPTO-MIT testing dataset, round-trip accuracy shows almost no difference. When reagent information is absent, the model reaches up to 87% round-trip accuracy with USPTO-50k (see Figure S10, Supporting Information), which is similar to the 88% obtained with the USPTO-MIT testing dataset (Figure 3, bottom). This suggests that, although top-1 accuracy flags some predictions of the reactant prediction model as incorrect because of discrepancies between datasets, round-trip accuracy considers that most of these predictions are valid alternatives to the targets present in the testing datasets. Even when the models are trained with reagents (which are absent from USPTO-50k), round-trip accuracy still reaches up to 80% (Figure S10, Supporting Information). Round-trip accuracy is validated by chemistry experts in Section 3.5.

### 3.3 Analysis of Reagent Predictions

**Figure 4:**
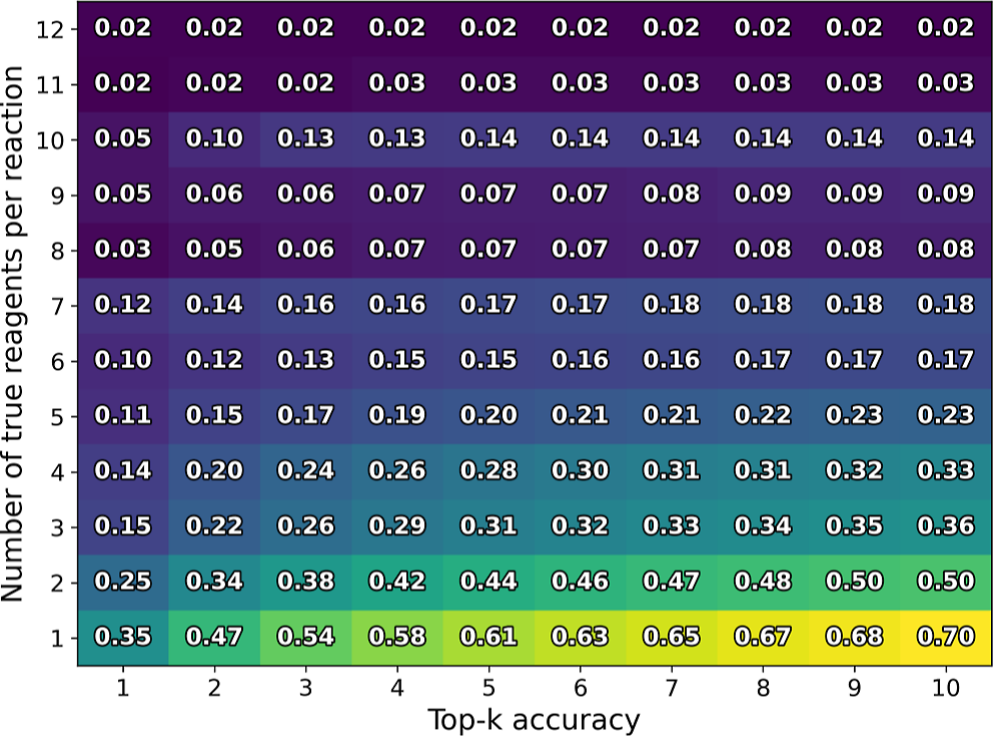
Top-*k* accuracy for reagent prediction, binning reactions of the USPTO-MIT testing dataset by the number of target reagents. The model was trained using the original USPTO-MIT dataset. The result of models trained with augmented training data are shown in Figures S11–S14 (Supporting Information).

We investigated the reasons of the poorer performance of the molecular transformer for reagent prediction (Figure 2, red). One reason might be that the nature of the task is very different, since reagents have no atom mapped to the product because they only play a transient role in the reaction. However, another reason might be that the number of reagents per reaction is quite large and varies a lot across USPTO-MIT. Indeed, the number of reagents per reaction ranges from 0 to 21 (median = 3, mean = 3.03, and std = 2.22), whereas the number of reactants ranges from 1 to 5 (median = 2, mean = 1.78, and std = 0.47). For this reason, we conducted an in-depth analysis of reagent prediction, monitoring the performance of the reagent prediction model for different subsets of the USPTO-MIT testing dataset and binning reactions by the number of reagents they contain. We evaluated the model that was trained with the original training dataset (no data augmentation) as it reached the best accuracy. In general, the predictive performance of the model is worse for reactions that require many reagents and better for reactions with few reagents (Figure 4).

**Figure 5:**
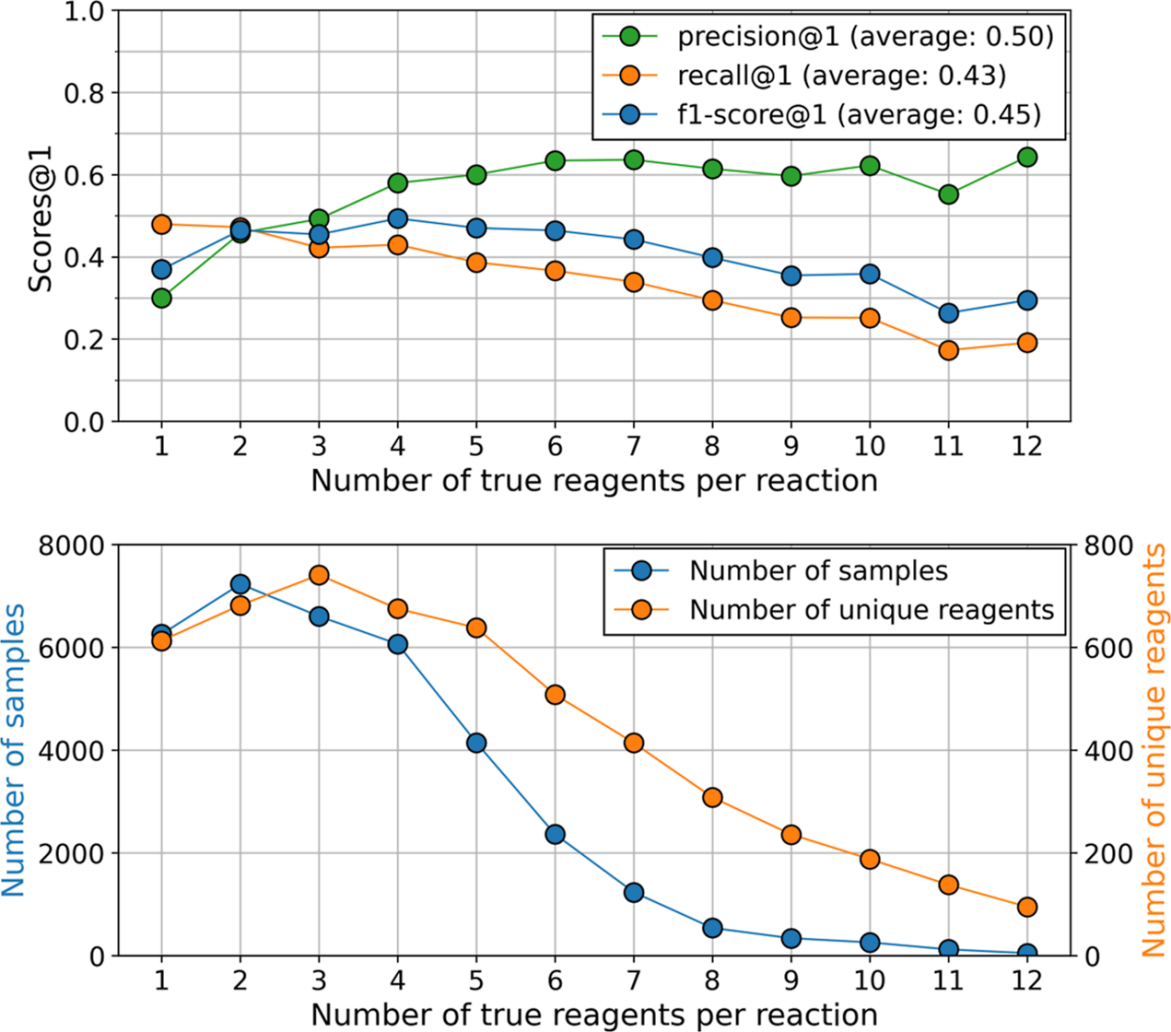
Statistical analysis of reagent prediction with the molecular transformer. Top. Precision, recall, and f1-score at rank 1 for reagent predictions, grouping samples of the USPTO-MIT test dataset by the number of target reagents they contain. Bottom. Number of reactions (blue line) and number of different reagent species (orange line) for each group of reactions. Chemical reactions with more than 12 reagents were discarded because their volume is not sufficient.

Then, we carried out an analysis of the model reagent predictions at the molecule level. Figure 5 (top) shows precision, recall, and f1-score at rank 1 for the same subsets of the testing dataset as in Figure 4. More details about how these metrics were computed are included in Section 2.4. The results of this analysis for different ranks (*k* > 1) are shown in Figures S15–S18 (Supporting Information). Average scores were computed by pooling the scores of each group of reactions, weighting them by the number of reactions per group.

We observe an inverted U-shape for the f1-score, which is the result of a regular increase of precision and a regular decrease of recall, with the number of reagents (see Section 2.4 for the formulas). This suggests that, for reactions with few reagents, although it is easier to recall the target reagent(s), the model is confused by the large number of possible reagent species (see Figure 5, bottom), which has a detrimental effect on precision. For reactions with many reagents, the model might no longer be able to identify all reagents necessary for the reactions (low recall) because there are too many target molecules and not enough training samples (see Figure 5, bottom). Nevertheless, the model still reaches a precision score of above 60% at rank 1 for reactions with more than five reagents per reaction (see Figure 5, top). This means that, even though the model was trained with a minority of reactions with many reagents (see Figure 5, bottom), 60% of the proposed reagents are correct.

### 3.4 Input Format, Tokenization, and Embeddings

We trained the model for all tasks described in Section 2.1 using the original dataset (i.e., no data augmentation), for all combinations of molecule formats, tokenization schemes, and embedding strategies described in Section 2.3. Model performance on the USPTO-MIT testing dataset is shown in Table 1. Results with the USPTO-50k dataset and for top-*k* accuracy (*k* > 1) are included in Tables S1–S8 (Supporting Information).

**Table 1:** Top-1 Accuracy Using Different Molecule Formats, Tokenization Schemes, and Embeddings Strategies^a^

	atom-Level		BPE	
	FS	PT	FS	PT
Product Prediction (with Reagents)				
SMILES	**0.879**	0.865	0.854	0.512
SELFIES	0.768	0.721	0.654	0.313
Product Prediction (without Reagents)				
SMILES	**0.837**	0.827	0.807	0.589
SELFIES	0.745	0.695	0.623	0.379
Reactant Prediction (with Reagents)				
SMILES	**0.678**	0.643	0.660	0.421
SELFIES	0.610	0.545	0.540	0.301
Reactant Prediction (without Reagent)				
SMILES	**0.525**	0.504	0.514	0.401
SELFIES	0.472	0.449	0.427	0.311
Reagent Prediction				
SMILES	0.196	0.135	0.183	**0.211**
SELFIES	0.187	0.122	0.174	0.196

a FS—input embeddings trained from scratch, and PT—pre-trained input embeddings.

For all tasks involving product or reactant prediction, the best model performance is obtained for the combination of the SMILES format, atom-level tokenization, and input embeddings trained from scratch (see Table 1, bold). The lowest model performance is obtained with the combination of the SELFIES format, BPE tokenization, and pre-trained embeddings. Intermediate values are found for different combinations. More precisely, enabling any of the latter input settings is detrimental for model performance: using the SELFIES format is always worse than using the SMILES format, using BPE tokenization is always worse than using atom-level tokenization, and using pre-trained embeddings is always worse than using embeddings trained from scratch.

For comparison, a recent study^67^ showed that BPE tokenization reached worse or similar reactant prediction performance as compared to atom-based tokenization, even with small vocabulary sizes (i.e., smaller than 100). Another study^68^ found that using SMILES slightly outperforms SELFIES for retrosynthesis and attributed it to the larger average length of the SELFIES string. Finally, using a BERT model pre-trained on PubChem^69^ compounds, ref (70) showed that classification performance on MoleculeNet^71^ tasks slightly worsened with BPE tokenization, with a vocabulary size of 1866, and did not improve with the SELFIES format.

Although different vocabulary sizes and differences in input lengths could explain these results, we speculate that the reason, as stated in the Introduction section, is how stable or expressive a model becomes when trained with different input settings. Although SELFIES increases input length, BPE tokenization shortens it, and using pre-trained embedding has no effect on input length. Still, all of them lead to worse model performance for product and reactant prediction tasks, for which the ability to generate a rich set of different molecules is crucial.

For reagent prediction, however, although the combination of the SMILES format, atom-level tokenization, and input embeddings trained from scratch provides almost the best model performance (20% top-1 accuracy), other combinations slightly outperform these settings. Notably, the combination of the SMILES format, BPE tokenization, and pre-trained input embeddings reaches 21% top-1 accuracy. Again, we speculate that this might be explained by the trade-off between input stability and expressivity. In reagent prediction, generating a rich set of molecules is less crucial, since there are fewer reagent species in USPTO-MIT (12k in the training dataset and 13.4k in the whole dataset), compared to products and reactants. This favors more stable settings. Besides, reagent molecule strings are shorter than reactants and products, and expressing reagents using BPE tokenization requires only a few tokens, which may be beneficial during training.

### 3.5 Validation of Round-Trip Analysis with Human Experts

**Figure 6:**
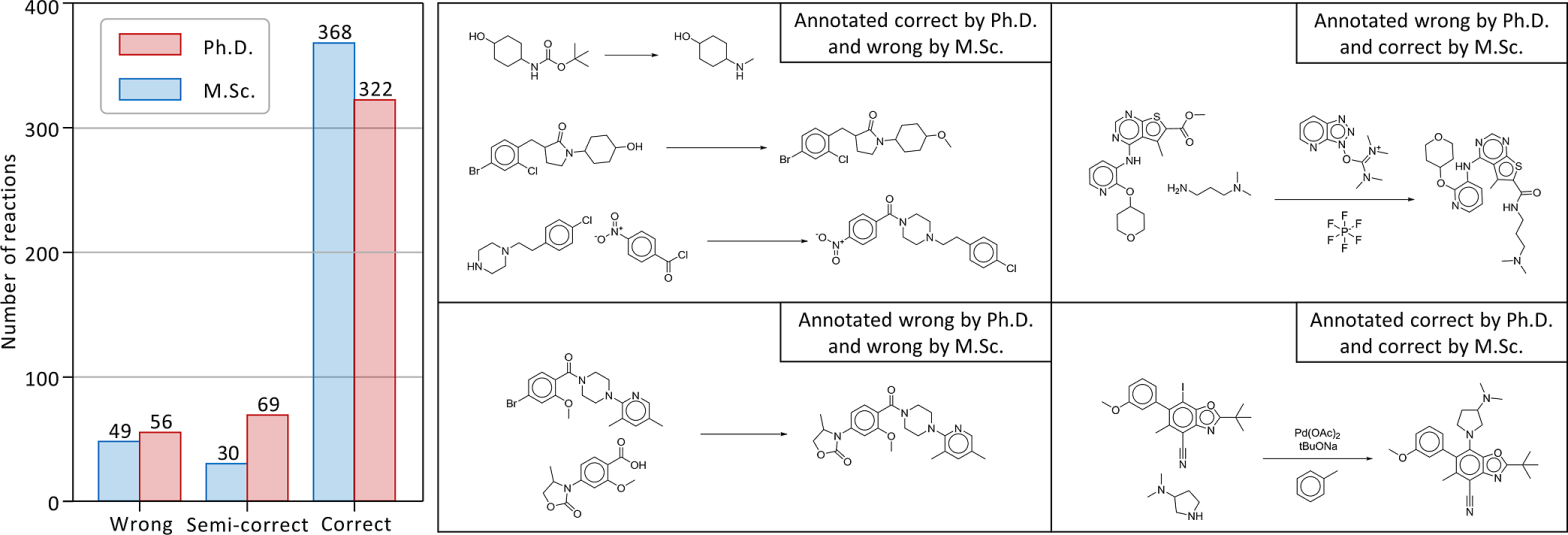
Round-trip validation by a committee of chemistry experts. Left. Number of reactions judged as wrong, semi-correct, and correct by the master and PhD groups. Right. Examples of reactions for which the master and doctorate groups agreed or disagreed.

Although the model reaches high round-trip accuracy scores in the reactant prediction tasks (see Section 3.2), it is still possible that the chemical reactions suggested by the round-trip analysis are not physically consistent. Therefore, to confirm the validity of the round-trip analysis, we cross-checked 447 randomly selected reactions whose predicted reactants did not match the target compounds of the USPTO-MIT dataset but succeeded in the round-trip experiment. As described in Section 2.4, we asked two groups of experts (master’s degree, PhD degree) to evaluate these reactions. The results of these evaluation are shown in Figure 6. In total, 81% (*n* = 362) of the reactions were classified as “not wrong”, i.e., either “correct” or “semi-correct”, by both groups of experts. Results were similar for both groups taken separately (89% for the master group and 87% for the doctorate group, as shown in Figure 6, left).

An example of reaction deemed correct by both groups is shown in Figure 6 (right, bottom-right quadrant), where the palladium-catalyzed coupling of the amine and aromatic iodine (Buchwald–Hartwig) reaction was recognized and confirmed by both expert groups. Only 4% (*n* = 18) of the valid round-trip pathways were judged as incorrect by both groups. An example of such a reaction is shown in Figure 6 (right, bottom-left quadrant), in which the algorithm made a clear mistake by duplicating a part of the reactants. Finally, 15% (*n* = 67) of predicted pathways had contradictory judgments. Examples of such reactions are shown in Figure 6 (right, top-left quadrant), where a member of the PhD group correctly identified that a Boc protecting group can result in methylamine in strongly reducing conditions, and Figure 6 (right, top-right quadrant), where the peptic coupling would require a saponification step before being valid.

Overall, these results confirm that the reactant prediction performance of the molecular transformer is higher when validated for alternative retrosynthetic pathways, such as in the round-trip analysis. This highlights the importance of using a broader set of metrics and evaluation benchmarks apart from direct comparisons to a predefined database, which may include a limited number of chemical reaction pathways. Given these results, we could have also considered the subset of reaction predictions that passed neither the round-trip test nor the simple accuracy test. Some of these reactions may be judged as valid by chemistry experts. Moreover, this experiment could have been extended to more recent models of chemical reaction prediction, such as ref (72), and increase the validity of the current results. We leave the investigation of such scenarios for future work.

## Conclusions

In this work, we performed detailed analyses of the capabilities of transformer models for chemical reaction prediction, using various predictive scenarios and input settings. We showed that data augmentation is not beneficial in all prediction scenarios and that its impact on performance depends on the metric that is used to evaluate the model. Moreover, we showed that using more stable but less expressive input settings might not always lead to better performance, which should be considered when choosing the type of embeddings, tokenization, and data format for the task of interest. Finally, we show that, for complex predictive scenarios such as retrosynthesis prediction, more elaborate evaluation metrics such as the round-trip analysis show better agreement with chemical experts than simple metrics based on the mere data present in the evaluation benchmark.

## Data and Software Availability

The data and code used to produce the results presented in this study are available at https://github.com/albornet/chempred_revision.

## Supporting Information Available

Results of Figure 2 but using top-*k* accuracy (*k* > 1) as an evaluation metric and using USPTO-50k as a testing dataset; analysis of Figure 3 but using USPTO-50k to compute round-trip accuracy; results of Figure 4 but with different data augmentation levels; results of Figure 5 but using different ranks (*k* > 1) for precision, recall, and f1-score; results of Table 1 but using top-*k* accuracy (*k* > 1) as an evaluation metric and using USPTO-50k as a testing dataset; and the very few molecules of the USPTO-MIT dataset that could not be encoded by the SELFIES encoder (PDF)

## Abbreviations

NLP: natural language processing
NMT: neural machine translator
RNN: recurrent neural network
LSTM: long short-term memory
SMILES: simplified molecular-input line-entry system
SELFIES: self-referencing embedded strings
USPTO: United States patent and trademark office
SPE: SMILES pair encoding

## References

[ref1] Scott D. E.; Bayly A. R.; Abell C.; Skidmore J. Small Molecules, Big Targets: Drug Discovery Faces the Protein–Protein Interaction Challenge. Nat. Rev. Drug Discovery 2016, 15, 533–550. 10.1038/nrd.2016.29. 27050677

[ref2] Beck H.; Härter M.; Haß B.; Schmeck C.; Baerfacker L. Small Molecules and Their Impact in Drug Discovery: A Perspective on the Occasion of the 125th Anniversary of the Bayer Chemical Research Laboratory. Drug Discovery Today 2022, 27, 1560–1574. 10.1016/j.drudis.2022.02.015. 35202802

[ref3] Ertl P. Cheminformatics Analysis of Organic Substituents: Identification of the Most Common Substituents, Calculation of Substituent Properties, and Automatic Identification of Drug-like Bioisosteric Groups. J. Chem. Inf. Comput. Sci. 2003, 43, 374–380. 10.1021/ci0255782. 12653499

[ref4] Bohacek R. S.; McMartin C.; Guida W. C. The Art and Practice of Structure-based Drug Design: A Molecular Modeling Perspective. Med. Res. Rev. 1996, 16, 3–50. 10.1002/(sici)1098-1128(199601)16:1<3::aid-med1>3.0.co;2-6. 8788213

[ref5] Polishchuk P. G.; Madzhidov T. I.; Varnek A. Estimation of the Size of Drug-like Chemical Space Based on GDB-17 Data. J. Comput.-Aided Mol. Des. 2013, 27, 675–679. 10.1007/s10822-013-9672-4. 23963658

[ref6] Garay-Ruiz D.; Álvarez-Moreno M.; Bo C.; Martínez-Núñez E. New Tools for Taming Complex Reaction Networks: The Unimolecular Decomposition of Indole Revisited. ACS Phys. Chem. Au 2022, 2, 225–236. 10.1021/acsphyschemau.1c00051. 36855573 PMC9718323

[ref7] Yang X.; Wang Y.; Byrne R.; Schneider G.; Yang S. Concepts of Artificial Intelligence for Computer-Assisted Drug Discovery. Chem. Rev. 2019, 119, 10520–10594. 10.1021/acs.chemrev.8b00728. 31294972

[ref8] Huang B.; von Lilienfeld O. A. Ab Initio Machine Learning in Chemical Compound Space. Chem. Rev. 2021, 121, 10001–10036. 10.1021/acs.chemrev.0c01303. 34387476 PMC8391942

[ref9] Butler K. T.; Davies D. W.; Cartwright H.; Isayev O.; Walsh A. Machine Learning for Molecular and Materials Science. Nature 2018, 559, 547–555. 10.1038/s41586-018-0337-2. 30046072

[ref10] Segler M. H. S.; Preuss M.; Waller M. P. Planning Chemical Syntheses with Deep Neural Networks and Symbolic AI. Nature 2018, 555, 604–610. 10.1038/nature25978. 29595767

[ref11] Gao H.; Struble T. J.; Coley C. W.; Wang Y.; Green W. H.; Jensen K. F. Using Machine Learning To Predict Suitable Conditions for Organic Reactions. ACS Cent. Sci. 2018, 4, 1465–1476. 10.1021/acscentsci.8b00357. 30555898 PMC6276053

[ref12] Patronov A.; Papadopoulos K.; Engkvist O. Has Artificial Intelligence Impacted Drug Discovery?. Methods Mol. Biol. 2022, 2390, 153–176. 10.1007/978-1-0716-1787-8_6. 34731468

[ref13] Sanchez-Lengeling B.; Aspuru-Guzik A. Inverse Molecular Design Using Machine Learning: Generative Models for Matter Engineering. Science 2018, 361, 360–365. 10.1126/science.aat2663. 30049875

[ref14] Mahmood O.; Mansimov E.; Bonneau R.; Cho K. Masked Graph Modeling for Molecule Generation. Nat. Commun. 2021, 12, 3156 10.1038/s41467-021-23415-2. 34039973 PMC8155025

[ref15] Schwaller P.; Vaucher A. C.; Laino T.; Reymond J.-L. Prediction of Chemical Reaction Yields Using Deep Learning. Mach. learn.: sci. technol. 2021, 2, 015016 10.1088/2632-2153/abc81d.

[ref16] Schwaller P.; Probst D.; Vaucher A. C.; Nair V. H.; Kreutter D.; Laino T.; Reymond J.-L. Mapping the Space of Chemical Reactions Using Attention-Based Neural Networks. Nat. Mach. Intell. 2021, 3, 144–152. 10.1038/s42256-020-00284-w.

[ref17] Probst D.; Schwaller P.; Reymond J.-L. Reaction Classification and Yield Prediction Using the Differential Reaction Fingerprint DRFP. Digit. Discovery 2022, 1, 91–97. 10.1039/D1DD00006C. PMC8996827 35515081

[ref18] Schwaller P.; Laino T.; Gaudin T.; Bolgar P.; Hunter C. A.; Bekas C.; Lee A. A. Molecular Transformer: A Model for Uncertainty-Calibrated Chemical Reaction Prediction. ACS Cent. Sci. 2019, 5, 1572–1583. 10.1021/acscentsci.9b00576. 31572784 PMC6764164

[ref19] Coley C. W.; Jin W.; Rogers L.; Jamison T. F.; Jaakkola T. S.; Green W. H.; Barzilay R.; Jensen K. F. A Graph-Convolutional Neural Network Model for the Prediction of Chemical Reactivity. Chem. Sci. 2019, 10, 370–377. 10.1039/C8SC04228D. 30746086 PMC6335848

[ref20] Fujinami M.; Seino J.; Nakai H. Quantum Chemical Reaction Prediction Method Based on Machine Learning. Bull. Chem. Soc. Jpn. 2020, 93, 685–693. 10.1246/bcsj.20200017.

[ref21] Segler M. H. S.; Waller M. P. Neural-Symbolic Machine Learning for Retrosynthesis and Reaction Prediction. Chem.—Eur. J. 2017, 23, 5966–5971. 10.1002/chem.201605499. 28134452

[ref22] Lee A. A.; Yang Q.; Sresht V.; Bolgar P.; Hou X.; Klug-McLeod J. L.; Butler C. R. Molecular Transformer Unifies Reaction Prediction and Retrosynthesis across Pharma Chemical Space. Chem. Commun. 2019, 55, 12152–12155. 10.1039/C9CC05122H. 31497831

[ref23] Schwaller P.; Petraglia R.; Zullo V.; Nair V. H.; Haeuselmann R. A.; Pisoni R.; Bekas C.; Iuliano A.; Laino T. Predicting Retrosynthetic Pathways Using Transformer-Based Models and a Hyper-Graph Exploration Strategy. Chem. Sci. 2020, 11, 3316–3325. 10.1039/C9SC05704H. 34122839 PMC8152799

[ref24] Tetko I. V.; Karpov P.; Van Deursen R.; Godin G. State-of-the-Art Augmented NLP Transformer Models for Direct and Single-Step Retrosynthesis. Nat. Commun. 2020, 11, 5575 10.1038/s41467-020-19266-y. 33149154 PMC7643129

[ref25] Chen S.; Jung Y. Deep Retrosynthetic Reaction Prediction Using Local Reactivity and Global Attention. JACS Au 2021, 1, 1612–1620. 10.1021/jacsau.1c00246. 34723264 PMC8549044

[ref26] Leguy J.; Cauchy T.; Glavatskikh M.; Duval B.; Da Mota B. EvoMol: A Flexible and Interpretable Evolutionary Algorithm for Unbiased de Novo Molecular Generation. J. Cheminf. 2020, 12, 55 10.1186/s13321-020-00458-z. PMC7494000 33431049

[ref27] Ahneman D. T.; Estrada J. G.; Lin S.; Dreher S. D.; Doyle A. G. Predicting Reaction Performance in C–N Cross-Coupling Using Machine Learning. Science 2018, 360, 186–190. 10.1126/science.aar5169. 29449509

[ref28] Schwaller P.; Hoover B.; Reymond J.-L.; Strobelt H.; Laino T. Extraction of Organic Chemistry Grammar from Unsupervised Learning of Chemical Reactions. Sci. Adv. 2021, 7, eabe4166 10.1126/sciadv.abe4166. 33827815 PMC8026122

[ref29] Yi H.-C.; You Z.-H.; Huang D.-S.; Kwoh C. K. Graph Representation Learning in Bioinformatics: Trends, Methods and Applications. Briefings Bioinf. 2022, 23, bbab340 10.1093/bib/bbab340. 34471921

[ref30] Weininger D. SMILES, a Chemical Language and Information System. 1. Introduction to Methodology and Encoding Rules. J. Chem. Inf. Model. 1988, 28, 31–36. 10.1021/ci00057a005.

[ref31] Krenn M.; Häse F.; Nigam A.; Friederich P.; Aspuru-Guzik A. Self-Referencing Embedded Strings (SELFIES): A 100% Robust Molecular String Representation. Mach. learn.: sci. technol. 2020, 1, 045024 10.1088/2632-2153/aba947.

[ref32] O’Boyle N.; Dalke A. DeepSMILES: An Adaptation of SMILES for Use in Machine-Learning of Chemical Structures. ChemRxiv 2018, 10.26434/chemrxiv.7097960.v1.

[ref33] O’Boyle N. M. Towards a Universal SMILES Representation-A Standard Method to Generate Canonical SMILES Based on the InChI. J. Cheminf. 2012, 4, 22 10.1186/1758-2946-4-22. PMC3495655 22989151

[ref34] Heller S.; McNaught A.; Stein S.; Tchekhovskoi D.; Pletnev I. InChI-the Worldwide Chemical Structure Identifier Standard. J. Cheminf. 2013, 5, 7 10.1186/1758-2946-5-7. PMC3599061 23343401

[ref35] Krenn M.; Ai Q.; Barthel S.; Carson N.; Frei A.; Frey N. C.; Friederich P.; Gaudin T.; Gayle A. A.; Jablonka K. M. SELFIES and the Future of Molecular String Representations. 2022, arXiv Prepr. arXiv220400056. 10.1016/j.patter.2022.100588 PMC9583042 36277819

[ref36] Jawahar G.; Sagot B.; Seddah D. What Does BERT Learn about the Structure of Language?. Proceedings of the 57th Annual Meeting of the Association for Computational Linguistics; Association for Computational Linguistics: Stroudsburg, PA, USA, 2019; pp 3651–3657.

[ref37] Yenicelik D.; Schmidt F.; Kilcher Y. How Does BERT Capture Semantics? A Closer Look at Polysemous Words. Proceedings of the Third BlackboxNLP Workshop on Analyzing and Interpreting Neural Networks for NLP; Association for Computational Linguistics: Stroudsburg, PA, USA, 2020; pp 156–162.

[ref38] Lowe D. M. Extraction of Chemical Structures and Reactions from the Literature, Ph.D. Thesis, University of Cambridge, 2012.

[ref39] He J.; Quoc Nguyen D.; Akhondi S. A.; Druckenbrodt C.; Thorne C.; Hoessel R.; Afzal Z.; Zhai Z.; Fang B.; Yoshikawa H.; Albahem A.; Wang J.; Ren Y.; Zhang Z.; Zhang Y.; Hoang Dao M.; Ruas P.; Lamurias A.; Couto F. M.; Copara Zea J. L.; Naderi N.; Knafou J. D. M.; Ruch P.; Teodoro D.; Lowe D. M.; Mayfield J.; Köksal A.; Dönmez H.; Özkirimli E.; Özgür A.; Mahendran D.; Gurdin G.; Lewinski N.; Tang C.; McInnes B. T.; Malarkodi C. S.; Rk Rao P.; Lalitha Devi S.; Cavedon L.; Cohn T.; Baldwin T.; Verspoor K. An Extended Overview of the CLEF 2020 ChEMU Lab: Information Extraction of Chemical Reactions from Patents. Proceedings of CLEF (Conference and Labs of the Evaluation Forum) 2020 Working Notes; CEUR Workshop Proceedings (CEUR-WS.org): Thessaloniki, Greece, 2020.

[ref40] Lowe D. M. Chemical Reactions from US Patents (1976-Sep2016). Figshare Dataset, 2017. 10.6084/m9.figshare.5104873.v1.

[ref41] Murakami Y.; Shono A. Reaction Engineering with Recurrent Neural Network: Kinetic Study of Dushman Reaction. Chem. Eng. J. Adv. 2022, 9, 100219 10.1016/j.ceja.2021.100219.

[ref42] Bort W.; Baskin I. I.; Gimadiev T.; Mukanov A.; Nugmanov R.; Sidorov P.; Marcou G.; Horvath D.; Klimchuk O.; Madzhidov T.; Varnek A. Discovery of Novel Chemical Reactions by Deep Generative Recurrent Neural Network. Sci. Rep. 2021, 11, 3178 10.1038/s41598-021-81889-y. 33542271 PMC7862614

[ref43] Vaswani A.; Shazeer N.; Parmar N.; Uszkoreit J.; Jones L.; Gomez A. N.; Kaiser Ł.; Polosukhin I. Attention Is All You Need. 31st Conference on Neural Information Processing Systems (NIPS 2017), 2017; pp 5998–6008.

[ref44] Lin T.; Wang Y.; Liu X.; Qiu X. A Survey of Transformers. 2021, arXiv Prepr. arXiv210604554.

[ref45] Duan H.; Wang L.; Zhang C.; Guo L.; Li J. Retrosynthesis with attention-based NMT model and chemical analysis of “wrong” predictions. RSC Adv. 2020, 10, 1371–1378. 10.1039/c9ra08535a. 35494683 PMC9047528

[ref46] Karpov P.; Godin G.; Tetko I. V. A Transformer Model for Retrosynthesis. International Conference on Artificial Neural Networks; Springer, 2019; pp 817–830.

[ref47] Zheng S.; Rao J.; Zhang Z.; Xu J.; Yang Y. Predicting Retrosynthetic Reactions Using Self-Corrected Transformer Neural Networks. J. Chem. Inf. Model. 2019, 60, 47–55. 10.1021/acs.jcim.9b00949. 31825611

[ref48] Bai R.; Zhang C.; Wang L.; Yao C.; Ge J.; Duan H. Transfer Learning: Making Retrosynthetic Predictions Based on a Small Chemical Reaction Dataset Scale to a New Level. Molecules 2020, 25, 2357 10.3390/molecules25102357. 32438572 PMC7287934

[ref49] Lin K.; Xu Y.; Pei J.; Lai L. Automatic Retrosynthetic Pathway Planning Using Template-Free Models. 2019, arXiv Prepr. arXiv190602308. 10.1039/c9sc03666k PMC8152431 34122843

[ref50] Schwaller P.; Probst D.; Vaucher A. C.; Nair V. H.; Laino T.; Reymond J.-L. Data-Driven Chemical Reaction Classification, Fingerprinting and Clustering Using Attention-Based Neural Networks. 2019, ChemRxiv: 10.26434/chemrxiv.9897365.v2.

[ref51] Fortunato M. E.; Coley C. W.; Barnes B. C.; Jensen K. F. Data Augmentation and Pretraining for Template-Based Retrosynthetic Prediction in Computer-Aided Synthesis Planning. J. Chem. Inf. Model. 2020, 60, 3398–3407. 10.1021/acs.jcim.0c00403. 32568548

[ref52] Sennrich R.; Haddow B.; Birch A. Neural Machine Translation of Rare Words with Subword Units. 2015, arXiv Prepr. arXiv150807909.

[ref53] Schuster M.; Nakajima K. Japanese and Korean Voice Search. 2012 IEEE international conference on acoustics, speech and signal processing (ICASSP); IEEE, 2012; pp 5149–5152.

[ref54] Kudo T.; Richardson J. Sentencepiece: A Simple and Language Independent Subword Tokenizer and Detokenizer for Neural Text Processing. 2018, arXiv Prepr. arXiv180806226.

[ref55] Summary of the tokenizers. https://huggingface.co/docs/transformers/tokenizer_summary (accessed Oct 12, 2022).

[ref56] Li X.; Fourches D. SMILES Pair Encoding: A Data-Driven Substructure Tokenization Algorithm for Deep Learning. J. Chem. Inf. Model. 2021, 61, 1560–1569. 10.1021/acs.jcim.0c01127. 33715361

[ref57] Socher R.; Bauer J.; Manning C. D.; Ng A. Y. Parsing with Compositional Vector Grammars. Proceedings of the 51st Annual Meeting of the Association for Computational Linguistics; Long Papers, 2013; Vol. 1, pp 455–465.

[ref58] Socher R.; Perelygin A.; Wu J.; Chuang J.; Manning C. D.; Ng A. Y.; Potts C. Recursive Deep Models for Semantic Compositionality over a Sentiment Treebank. Proceedings of the 2013 conference on empirical methods in natural language processing, 2013; pp 1631–1642.

[ref59] Mikolov T.; Chen K.; Corrado G.; Dean J. Efficient Estimation of Word Representations in Vector Space. 1st International Conference on Learning Representations; ICLR 2013—Workshop Track Proceedings, 2013.

[ref60] Linsley D.; Karkada Ashok A.; Govindarajan L. N.; Liu R.; Serre T. Stable and Expressive Recurrent Vision Models. Adv. Neural Inf. Process. Syst. 2020, 33, 10456–10467.

[ref61] Thakkar A.; Kogej T.; Reymond J.-L.; Engkvist O.; Bjerrum E. J. Datasets and Their Influence on the Development of Computer Assisted Synthesis Planning Tools in the Pharmaceutical Domain. Chem. Sci. 2020, 11, 154–168. 10.1039/c9sc04944d. 32110367 PMC7012039

[ref62] Klein G.; Kim Y.; Deng Y.; Senellart J.; Rush A. OpenNMT: Open-Source Toolkit for Neural Machine Translation. Proceedings of ACL 2017, System Demonstrations; Association for Computational Linguistics: Stroudsburg, PA, USA, 2017; pp 67–72.

[ref63] Jin W.; Coley C.; Barzilay R.; Jaakkola T. Predicting Organic Reaction Outcomes with Weisfeiler-Lehman Network. 31st Conference on Neural Information Processing Systems (NIPS 2017), 2017.

[ref64] Bradshaw J.; Kusner M.; Paige B.; Segler M.; Hernández-Lobato J. Generative Model For Electron Paths. ICLR: International Conference on Learning Representations, 2019.

[ref65] Schwaller P.; Gaudin T.; Lányi D.; Bekas C.; Laino T. “Found in Translation”: predicting outcomes of complex organic chemistry reactions using neural sequence-to-sequence models. Chem. Sci. 2018, 9, 6091–6098. 10.1039/c8sc02339e. 30090297 PMC6053976

[ref66] Liu B.; Ramsundar B.; Kawthekar P.; Shi J.; Gomes J.; Luu Nguyen Q.; Ho S.; Sloane J.; Wender P.; Pande V. Retrosynthetic Reaction Prediction Using Neural Sequence-to-Sequence Models. ACS Cent. Sci. 2017, 3, 1103–1113. 10.1021/acscentsci.7b00303. 29104927 PMC5658761

[ref67] Tran K. Optimization of Molecular Transformers: Influence of Tokenization Schemes. M.Sc. Thesis, Chalmers University of Technology, 2021.

[ref68] Sun R.; Dai H.; Li L.; Kearnes S.; Dai B. Energy-Based View of Retrosynthesis. 2020, arXiv Prepr. arXiv200713437.

[ref69] Kim S.; Thiessen P. A.; Bolton E. E.; Chen J.; Fu G.; Gindulyte A.; Han L.; He J.; He S.; Shoemaker B. A.; Wang J.; Yu B.; Zhang J.; Bryant S. H. PubChem Substance and Compound Databases. Nucleic Acids Res. 2016, 44, D1202–D1213. 10.1093/nar/gkv951. 26400175 PMC4702940

[ref70] Chithrananda S.; Grand G.; Ramsundar B. ChemBERTa: Large-Scale Self-Supervised Pretraining for Molecular Property Prediction. 2020, arXiv Prepr. arXiv201009885.

[ref71] Wu Z.; Ramsundar B.; Feinberg E. N.; Gomes J.; Geniesse C.; Pappu A. S.; Leswing K.; Pande V. MoleculeNet: A Benchmark for Molecular Machine Learning. Chem. Sci. 2018, 9, 513–530. 10.1039/c7sc02664a. 29629118 PMC5868307

[ref72] Chen D.; Zheng J.; Wei G.-W.; Pan F. Extracting Predictive Representations from Hundreds of Millions of Molecules. J. Phys. Chem. Lett. 2021, 12, 10793–10801. 10.1021/acs.jpclett.1c03058. 34723543 PMC9358546

