# Supplementary material for: Transformer Performance for Chemical Reactions: Analysis of Different Predictive and Evaluation Scenarios
Source: J Chem Inf Model. 2023 Mar 23;63(7):1914–24. doi: 10.1021/acs.jcim.2c01407 (PMC10091402; doi:10.1021/acs.jcim.2c01407)
Supplement: Supplementary file 1 — ci2c01407_si_001.pdf [file ci2c01407_si_001.pdf]

Transformer performance for chemical reactions: analysis of different predictive and evaluation scenarios

Fernando Jaume-Santero[‡1,2], Alban Bornet[‡1,2], Alain Valery[3], Nona Naderi[2,4], David Vicente Alvarez[1,2], Dimitrios Proios[1], Anthony Yazdani[1], Colin Bournez[3], Thomas Fessard[3], Douglas Teodoro[*1,2,4]

[1] Department of Radiology and Medical Informatics, University of Geneva, 1205 Geneva, Switzerland

[2] Geneva School of Business Administration, HES-SO University of Applied Sciences and Arts of Western Switzerland, 1227 Geneva, Switzerland

[3] SpiroChem AG, 4058 Basel, Switzerland

[4] Swiss Institute of Bioinformatics, 1015 Lausanne, Switzerland

[‡] Equal contribution

[*] Corresponding author - douglas.teodoro@unige.ch

S-1 – SUPPLEMENTARY INFORMATION FOR FIGURE 2 – TOP-K ACCURACY (K>=1) – USPTO-MIT

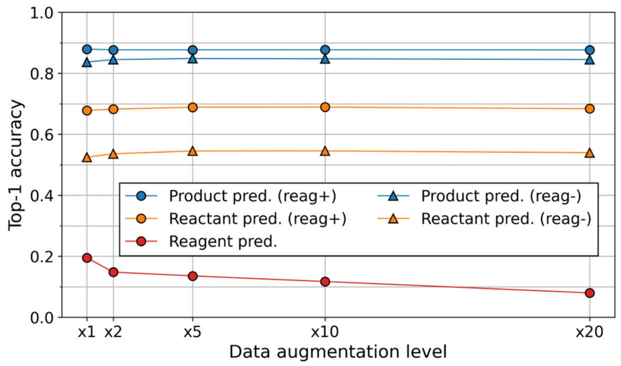

Figure S1. Top-1 accuracy for different data augmentation levels, using the USPTO-MIT testing dataset (same as Figure 2).

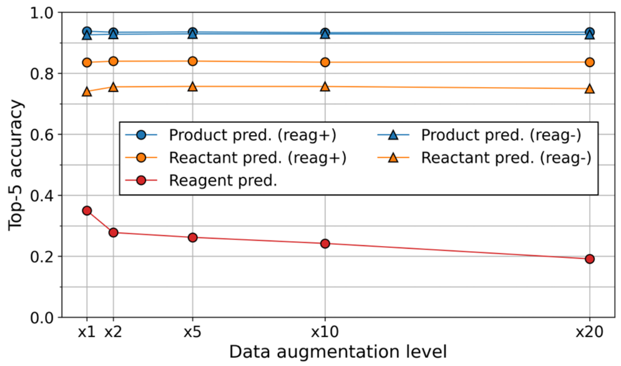

Figure S3. Top-5 accuracy for different data augmentation levels, using the USPTO-MIT testing dataset.

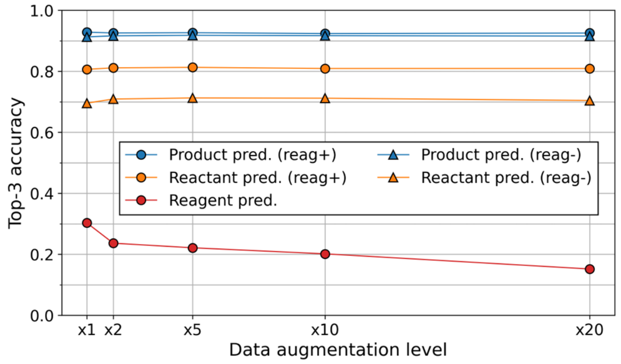

Figure S2. Top-3 accuracy for different data augmentation levels, using the USPTO-MIT testing dataset.

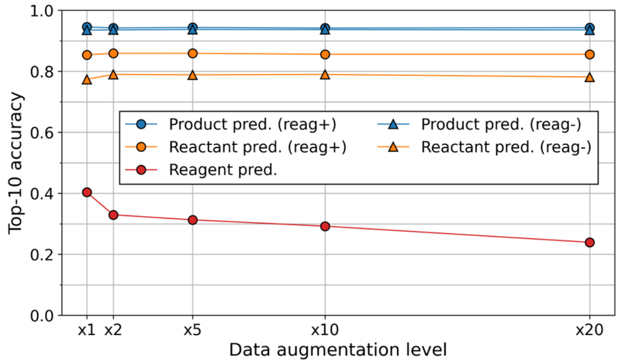

Figure S4. Top-10 accuracy for different data augmentation levels, using the USPTO-MIT testing dataset.

S-2 – SUPPLEMENTARY INFORMATION FOR FIGURE 2 – TOP-K ACCURACY (K>=1) – USPTO-50K

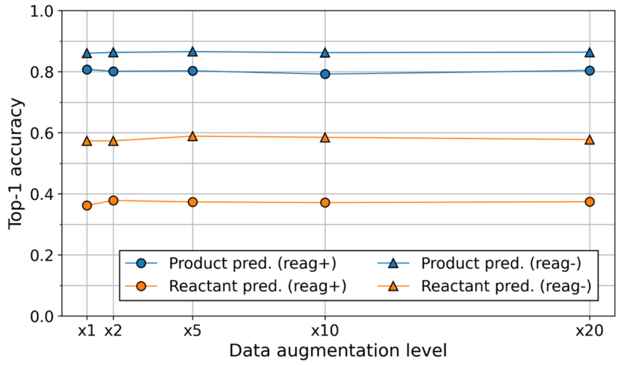

Figure S5. Top-1 accuracy for different data augmentation levels, using the USPTO-50k dataset.

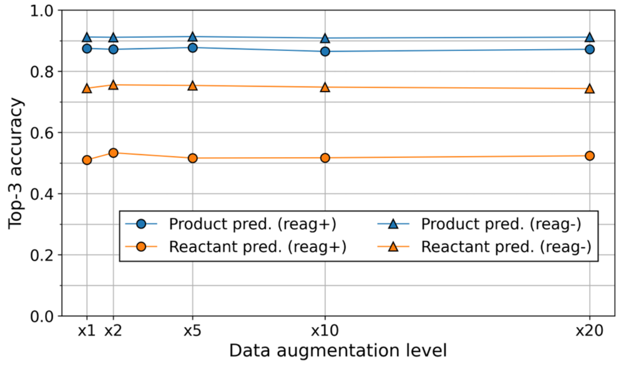

Figure S6. Top-3 accuracy for different data augmentation levels, using the USPTO-50k dataset.

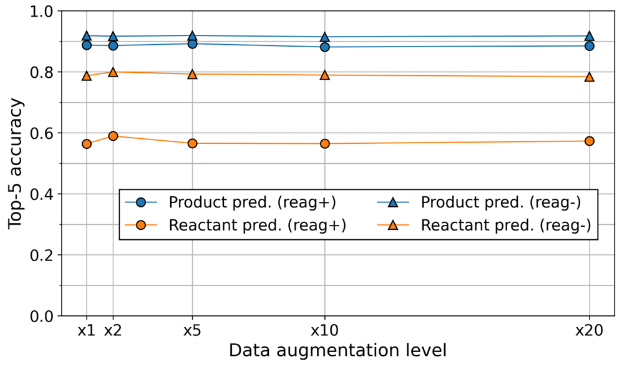

Figure S7. Top-5 accuracy for different data augmentation levels, using the USPTO-50k dataset.

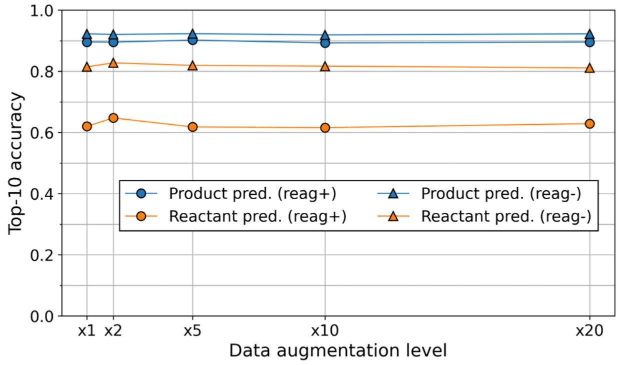

Figure S8. Top-10 accuracy for different data augmentation levels, using the USPTO-50k dataset.

S-3 – SUPPLEMENTARY INFORMATION FOR FIGURE 3 – USPTO-MIT AND USPTO-50K

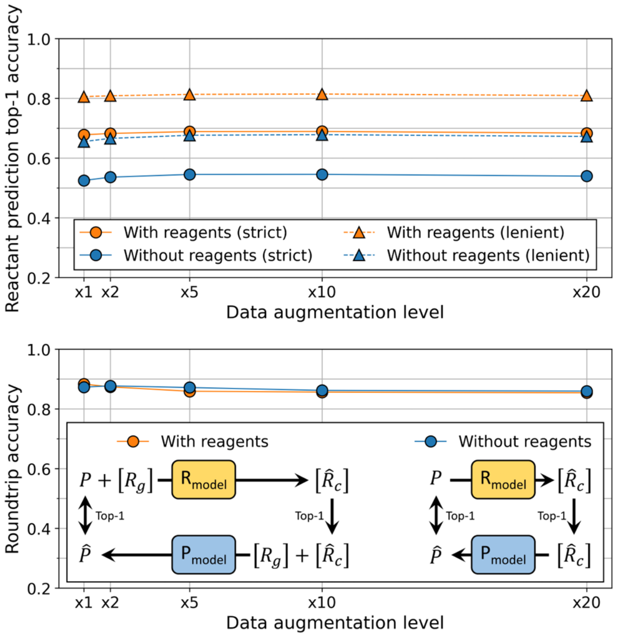

Figure S9. Same as Figure 3. Top-1 and round-trip accuracy for the reactant prediction task, using the USPTO-MIT testing dataset, for different levels of data augmentation. Top. Top-1 accuracy. “Strict” requires an exact match between the model prediction and the target. "Lenient” requires that at least one molecule predicted by the model matches a target molecule. Bottom. Round-trip accuracy. The diagram shows how round-trip accuracy was computed. When reagents were part of the datasets, the true reagents were added to the predicted reactants before being fed to the product prediction model. P – true product, $[R_c]$ – true reactant(s), $[R_g]$ – true reagent(s), $\hat{P}$ – predicted product, $[\hat{R}_c]$ – predicted reactant(s).

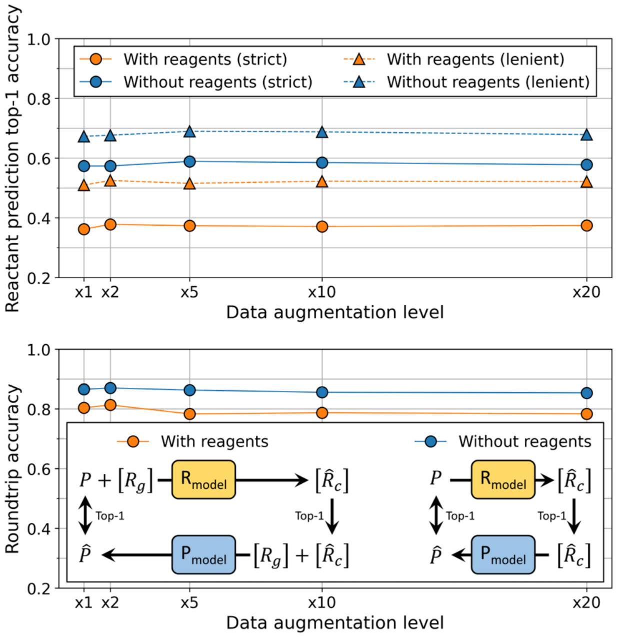

Figure S10. Top-1 and round-trip accuracy for the reactant prediction task, using the USPTO-50k testing dataset, for different levels of data augmentation. Top. Top-1 accuracy. “Strict” requires an exact match between the model prediction and the target. "Lenient” requires that at least one molecule predicted by the model matches a target molecule. Bottom. Round-trip accuracy. The diagram shows how round-trip accuracy was computed. When reagents were part of the datasets, the true reagents were added to the predicted reactants before they were fed to the product prediction model. P – true product, $[R_c]$ – true reactant(s), $[R_g]$ – true reagent(s), $\hat{P}$ – predicted product, $[\hat{R}_c]$ – predicted reactant(s). Note: lower performance is observed when the model was trained with reagent information, since no reagent information is included in the reactions of the USPTO-50k dataset. Still, the model reaches around 80% roundtrip accuracy.

S-4 – SUPPLEMENTARY INFORMATION FOR FIGURE 4 – DIFFERENT DATA AUGMENTATION LEVELS

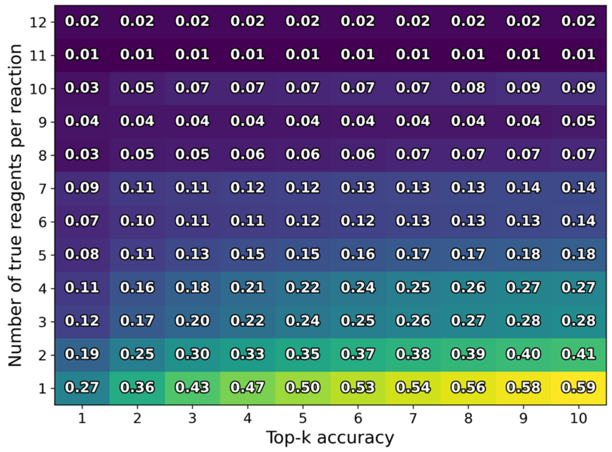

Figure S11. Top-k accuracy for reagent prediction, binning reactions of the USPTO-MIT testing dataset by the number of target reagents, after training the model with 2-fold data augmentation.

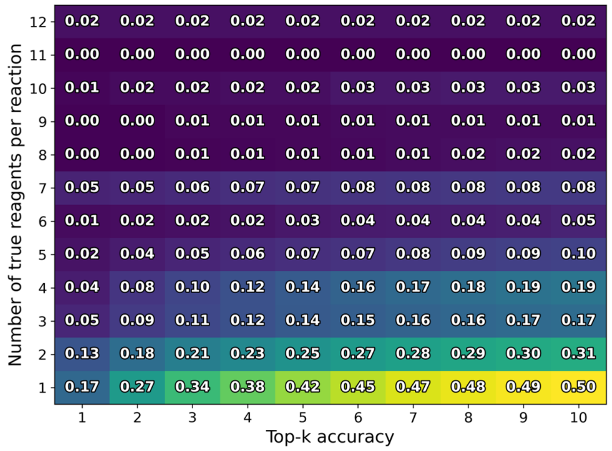

Figure S12. Top-k accuracy for reagent prediction, binning reactions of the USPTO-MIT testing dataset by the number of target reagents, after training the model with 5-fold data augmentation.

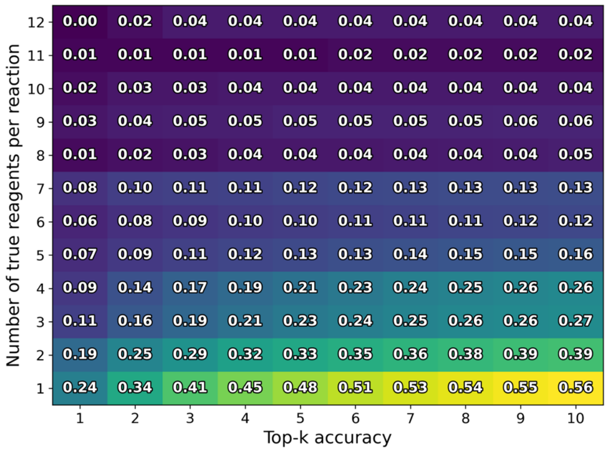

Figure S13. Top-k accuracy for reagent prediction, binning reactions of the USPTO-MIT testing dataset by the number of target reagents, after training the model with 10-fold data augmentation.

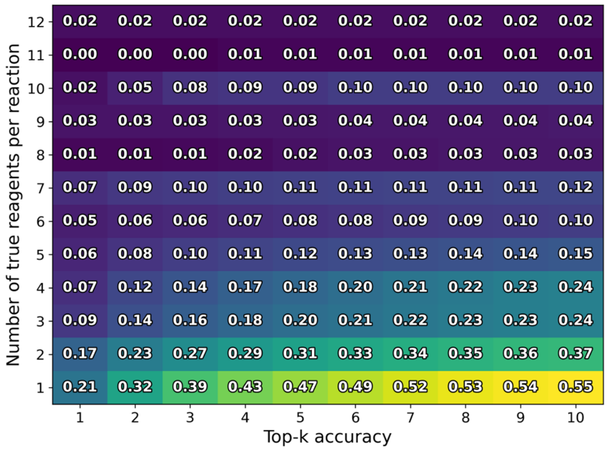

Figure S14. Top-k accuracy for reagent prediction, binning reactions of the USPTO-MIT testing dataset by the number of target reagents, after training the model with 20-fold data augmentation.

S-5 – SUPPLEMENTARY INFORMATION FOR FIGURE 5 – DIFFERENT RANKS (K>=1)

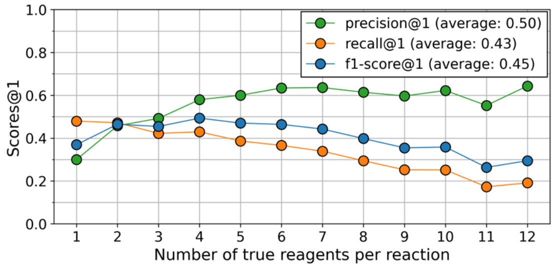

Figure S15. Same as Figure 5. Top. Precision, recall, and F1 scores at rank 1 for reagent predictions from the USPTO-MIT test dataset grouped by the number of target reagents.

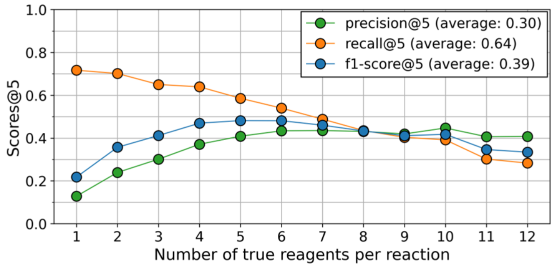

Figure S17. Top. Precision, recall, and F1 scores at rank 5 for reagent predictions from the USPTO-MIT test dataset grouped by the number of target reagents.

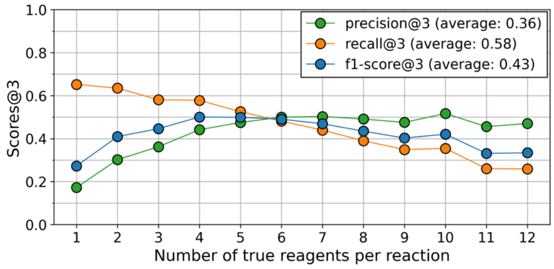

Figure S16. Precision, recall, and F1 scores at rank 3 for reagent predictions from the USPTO-MIT test dataset grouped by the number of target reagents.

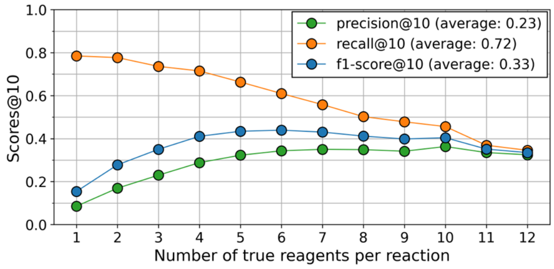

Figure S18. Top. Precision, recall, and F1 scores at rank 3 for reagent predictions from the USPTO-MIT test dataset grouped by the number of target reagents.

S-6 – SUPPLEMENTARY INFORMATION FOR TABLE 1 – TOP-K ACCURACY (K=1, 3) – USPTO-MIT

Table S1. Same as Table 1. Top-1 accuracy using different molecule formats, tokenization schemes and embeddings strategies. Models evaluated with USPTO-MIT testing data.

Product prediction (with reagents)

	Atom-level		BPE	
	FS	PT	FS	PT
SMILES	**0.879**	0.865	0.854	0.512
SELFIES	0.768	0.721	0.654	0.313

Product prediction (without reagents)

	Atom-level		BPE	
	FS	PT	FS	PT
SMILES	**0.837**	0.827	0.807	0.589
SELFIES	0.745	0.695	0.623	0.379

Reactant prediction (with reagents)

	Atom-level		BPE	
	FS	PT	FS	PT
SMILES	**0.678**	0.643	0.660	0.421
SELFIES	0.610	0.545	0.540	0.301

Reactant prediction (without reagent)

	Atom-level		BPE	
	FS	PT	FS	PT
SMILES	**0.525**	0.504	0.514	0.401
SELFIES	0.472	0.449	0.427	0.311

Reagent prediction

	Atom-level		BPE	
	FS	PT	FS	PT
SMILES	0.196	0.135	0.183	**0.211**
SELFIES	0.187	0.122	0.174	0.196

FS – input embeddings trained from scratch, PT – pre-trained input embeddings.

Table S2. Top-3 accuracy using different molecule formats, tokenization schemes and embeddings strategies. Models evaluated using USPTO-MIT testing datasets.

Product prediction (with reagents)

	Atom-level		BPE	
	FS	PT	FS	PT
SMILES	**0.928**	0.918	0.907	0.620
SELFIES	0.861	0.825	0.756	0.405

Product prediction (without reagents)

	Atom-level		BPE	
	FS	PT	FS	PT
SMILES	**0.913**	0.902	0.885	0.700
SELFIES	0.848	0.814	0.733	0.484

Reactant prediction (with reagents)

	Atom-level		BPE	
	FS	PT	FS	PT
SMILES	**0.806**	0.775	0.774	0.530
SELFIES	0.739	0.675	0.655	0.395

Reactant prediction (without reagent)

	Atom-level		BPE	
	FS	PT	FS	PT
SMILES	**0.696**	0.679	0.663	0.535
SELFIES	0.631	0.607	0.561	0.424

Reagent prediction

	Atom-level		BPE	
	FS	PT	FS	PT
SMILES	0.303	0.212	0.274	**0.314**
SELFIES	0.289	0.199	0.261	0.298

FS – input embeddings trained from scratch, PT – pre-trained input embeddings.

S-7 – SUPPLEMENTARY INFORMATION FOR TABLE 1 – TOP-K ACCURACY (K=5, 10) – USPTO-MIT

Table S3. Top-5 accuracy using different molecule formats, tokenization schemes and embeddings strategies. Models evaluated using USPTO-MIT testing datasets.

Product prediction (with reagents)

	Atom-level		BPE	
	FS	PT	FS	PT
SMILES	**0.938**	0.929	0.920	0.654
SELFIES	0.885	0.851	0.789	0.439

Product prediction (without reagents)

	Atom-level		BPE	
	FS	PT	FS	PT
SMILES	**0.926**	0.915	0.902	0.734
SELFIES	0.873	0.844	0.769	0.521

Reactant prediction (with reagents)

	Atom-level		BPE	
	FS	PT	FS	PT
SMILES	**0.836**	0.807	0.802	0.568
SELFIES	0.772	0.711	0.689	0.427

Reactant prediction (without reagent)

	Atom-level		BPE	
	FS	PT	FS	PT
SMILES	**0.741**	0.729	0.708	0.579
SELFIES	0.680	0.654	0.604	0.462

Reagent prediction

	Atom-level		BPE	
	FS	PT	FS	PT
SMILES	0.350	0.248	0.314	**0.357**
SELFIES	0.334	0.234	0.300	0.341

FS – input embeddings trained from scratch, PT – pre-trained input embeddings.

Table S4. Top-10 accuracy using different molecule formats, tokenization schemes and embeddings strategies. Models evaluated using USPTO-MIT testing datasets.

Product prediction (with reagents)

	Atom-level		BPE	
	FS	PT	FS	PT
SMILES	**0.945**	0.938	0.931	0.688
SELFIES	0.901	0.874	0.824	0.474

Product prediction (without reagents)

	Atom-level		BPE	
	FS	PT	FS	PT
SMILES	**0.935**	0.925	0.917	0.765
SELFIES	0.892	0.868	0.805	0.578

Reactant prediction (with reagents)

	Atom-level		BPE	
	FS	PT	FS	PT
SMILES	**0.854**	0.829	0.828	0.601
SELFIES	0.800	0.740	0.722	0.457

Reactant prediction (without reagent)

	Atom-level		BPE	
	FS	PT	FS	PT
SMILES	**0.774**	0.763	0.750	0.621
SELFIES	0.717	0.692	0.645	0.496

Reagent prediction

	Atom-level		BPE	
	FS	PT	FS	PT
SMILES	0.403	0.291	0.369	**0.416**
SELFIES	0.386	0.281	0.355	0.398

FS – input embeddings trained from scratch, PT – pre-trained input embeddings.

S-8 – SUPPLEMENTARY INFORMATION FOR TABLE 1 – TOP-K ACCURACY (K=1, 3) – USPTO-50K

Table S5. Top-1 accuracy using different molecule formats, tokenization schemes and embeddings strategies. Models evaluated using USPTO-50k. Note: The lower performance when the model was trained without reagent is explained by the absence of reagents in USPTO-50k.

Product prediction (with reagents)

	Atom-level		BPE	
	FS	PT	FS	PT
SMILES	**0.807**	0.790	0.733	0.374
SELFIES	0.693	0.654	0.548	0.235

Product prediction (without reagents)

	Atom-level		BPE	
	FS	PT	FS	PT
SMILES	**0.860**	0.851	0.835	0.631
SELFIES	0.774	0.728	0.682	0.482

Reactant prediction (with reagents)

	Atom-level		BPE	
	FS	PT	FS	PT
SMILES	**0.362**	0.349	0.360	0.207
SELFIES	0.332	0.294	0.305	0.156

Reactant prediction (without reagent)

	Atom-level		BPE	
	FS	PT	FS	PT
SMILES	0.573	0.549	**0.592**	0.484
SELFIES	0.508	0.489	0.525	0.401

FS – input embeddings trained from scratch, PT – pre-trained input embeddings.

Table S6. Top-3 accuracy using different molecule formats, tokenization schemes and embeddings strategies. Models evaluated using USPTO-50k. Note: The lower performance when the model was trained without reagent is explained by the absence of reagents in USPTO-50k.

Product prediction (with reagents)

	Atom-level		BPE	
	FS	PT	FS	PT
SMILES	**0.875**	0.869	0.812	0.447
SELFIES	0.802	0.770	0.641	0.306

Product prediction (without reagents)

	Atom-level		BPE	
	FS	PT	FS	PT
SMILES	**0.912**	0.904	0.889	0.700
SELFIES	0.854	0.830	0.756	0.623

Reactant prediction (with reagents)

	Atom-level		BPE	
	FS	PT	FS	PT
SMILES	**0.510**	0.482	0.472	0.285
SELFIES	0.461	0.409	0.394	0.216

Reactant prediction (without reagent)

	Atom-level		BPE	
	FS	PT	FS	PT
SMILES	**0.745**	0.731	0.709	0.594
SELFIES	0.671	0.648	0.648	0.626

FS – input embeddings trained from scratch, PT – pre-trained input embeddings.

S-9 – SUPPLEMENTARY INFORMATION FOR TABLE 1 – TOP-K ACCURACY (K=5, 10) – USPTO-50K

Table S7. Top-5 accuracy using different molecule formats, tokenization schemes and embeddings strategies. Models evaluated using USPTO-50k. Note: The lower performance when the model was trained without reagent is explained by the absence of reagents in USPTO-50k.

Product prediction (with reagents)

	Atom-level		BPE	
	FS	PT	FS	PT
SMILES	**0.887**	0.884	0.832	0.473
SELFIES	0.831	0.803	0.673	0.331

Product prediction (without reagents)

	Atom-level		BPE	
	FS	PT	FS	PT
SMILES	**0.919**	0.911	0.901	0.721
SELFIES	0.872	0.852	0.782	0.588

Reactant prediction (with reagents)

	Atom-level		BPE	
	FS	PT	FS	PT
SMILES	**0.564**	0.526	0.512	0.314
SELFIES	0.506	0.448	0.426	0.238

Reactant prediction (without reagent)

	Atom-level		BPE	
	FS	PT	FS	PT
SMILES	**0.787**	0.779	0.746	0.625
SELFIES	0.715	0.629	0.626	0.504

FS – input embeddings trained from scratch, PT – pre-trained input embeddings.

Table S8. Top-10 accuracy using different molecule formats, tokenization schemes and embeddings strategies. Models evaluated using USPTO-50k. Note: The lower performance when the model was trained without reagent is explained by the absence of reagents in USPTO-50k.

Product prediction (with reagents)

	Atom-level		BPE	
	FS	PT	FS	PT
SMILES	**0.896**	0.894	0.853	0.502
SELFIES	0.853	0.826	0.706	0.356

Product prediction (without reagents)

	Atom-level		BPE	
	FS	PT	FS	PT
SMILES	**0.922**	0.915	0.911	0.741
SELFIES	0.886	0.870	0.809	0.609

Reactant prediction (with reagents)

	Atom-level		BPE	
	FS	PT	FS	PT
SMILES	**0.620**	0.575	0.557	0.344
SELFIES	0.557	0.493	0.460	0.260

Reactant prediction (without reagent)

	Atom-level		BPE	
	FS	PT	FS	PT
SMILES	**0.815**	0.807	0.780	0.552
SELFIES	0.750	0.727	0.685	0.558

FS – input embeddings trained from scratch, PT – pre-trained input embeddings.

S-10 – MOLECULES WITHOUT SELFIES ENCODING

Table S9. SMILES molecules of the USPTO-MIT dataset that could not be encoded as SELFIES. Note: these molecules occurred very rarely in the reactions.

O=I(=O)Cl
Cl[IH2](Cl)Cl
O=[IH2]c1ccccc1
F[P-](F)(F)(F)(F)F
O=C(O)c1ccccc1I(=O)=O
O=C1OI(=O)(O)c2ccccc21
S=[Re](=S)(=S)(=S)(=S)(=S)=S
CC1(C)O[IH2](C(F)(F)F)c2ccccc21
C12C3C4C5C1[Fe]23451678C2C1C6C7C28
C12C3C4C5C1[Zr]23451678C2C1C6C7C28
O=C(OI(OC(=O)C(F)(F)F)c1ccccc1)C(F)(F)F
CC(=O)OI1(OC(C)=O)(OC(C)=O)OC(=O)c2ccccc21
Cc1ccc(S(=O)(=O)N=C2CCCC[IH2]2c2ccccc2)cc1
O=C(O[IH2](OC(=O)C(F)(F)F)c1ccccc1)C(F)(F)F
COc1cc2c(cc1OC)C([PH2](c1ccccc1)(c1ccccc1)c1ccccc1)OC2=O
O=c1[nH]c2c3occc3c(F)c(F)c2n1-c1ccc([IH]S(=O)(=O)C2CC2COCc2ccccc2)cc1F
